# Using a high density bin map to analyze quantitative trait locis of germination ability of maize at low temperatures

**DOI:** 10.3389/fpls.2022.978941

**Published:** 2022-08-22

**Authors:** Yu Zhou, Qing Lu, Jinxin Ma, Dandan Wang, Xin Li, Hong Di, Lin Zhang, Xinge Hu, Ling Dong, Xianjun Liu, Xing Zeng, Zhiqiang Zhou, Jianfeng Weng, Zhenhua Wang

**Affiliations:** ^1^Key Laboratory of Germplasm Enhancement, Physiology and Ecology of Food Crops in Cold Region, Ministry of Education, Northeast Agricultural University, Harbin, China; ^2^Institute of Crop Science, Chinese Academy of Agricultural Sciences, Beijing, China

**Keywords:** maize, low temperature, germination, high-density linkage map, candidate gene

## Abstract

Low temperatures in the spring often lead to a decline in the emergence rate and uniformity of maize, which can affect yield in northern regions. This study used 365 recombinant inbred lines (RILs), which arose from crossing Qi319 and Ye478, to identify low-temperature resistance during the germination stage by measuring eight low-temperature-related traits. The quantitative trait locis (QTLs) were mapped using *R/qtl* software by combining phenotypic data, and the genotyping by sequencing (GBS) method to produce a high-density genetic linkage map. Twenty QTLs were detected during QTL mapping, of which seven QTLs simultaneously detected a consistent 197.10–202.30 Mb segment on chromosome 1. The primary segment was named *cQTL1-2*, with a phenotypic variation of 5.18–25.96% and a physical distance of 5.2 Mb. This combines the phenotype and genotype with the identification of seven chromosome segment substitution lines (CSSLs), which were derived from Ye478*Qi319 and related to *cQTL1-2*. The physical distance of *cQTL1-2* was reduced to approximately 1.9 Mb. The consistent meta-QTL *mQTL1* was located at 619.06 cM on chromosome 1, had a genetic distance of 7.27 cM, and overlapped with *cQTL1-2*. This was identified by combining the results of previous QTL studies assessing maize tolerance to low temperatures at the germination stage. An assessment of the results of the RIL population, CSSLs, and *mQTL1* found the consistent QTL to be *LtQTL1-1*. It was identified in bin1.06-1.07 at a confidence interval of between 200,400,148 and 201,775,619 bp. In this interval, qRT-PCR found that relative expression of the candidate genes *GRMZM2G082630* and *GRMZM2G115730* were both up-regulated in low-temperature tolerant lines and down-regulated in sensitive lines (*P* < 0.01).

## Introduction

Cold temperatures can affect the development, biochemistry, physiology, productivity, and quality of plants, making it one of the primary factors limiting the distribution of plants worldwide (Kocsy et al., 2011; Szalai et al., 2018). Maize (*Zea mays* L.) is an important crop, representing 40% of global cereal production^1^ (Bouis and Welch, 2010). Maize is sensitive to cold temperatures, especially during its early growth, including the germination and seedling stages, because it originated in tropical and subtropical locations (Greaves, 1996; Verheul et al., 1996; Li et al., 2018).

When subjected to low temperatures, plant roots do not fully grow (Zhu et al., 2015). In *Arabidopsis thaliana*, growth of the main root is limited by low temperatures (Plohovska et al., 2016), while some genes, such as *CYTOKININ RESPONSE FACTOR2* (*CRF2*) and *CRF3*, serve vital functions and regulate the growth of the lateral roots of *A. thaliana* under low-temperature conditions (Jeon et al., 2016; Rativa et al., 2020). Some genes in the shoots and roots of maize and rice, such as *Adhl*, show a rapid increase in steady-state levels when exposed to low temperatures (Christie et al., 2021). The key issue affecting the ability of maize to germinate at low temperatures is appropriately identifying its phenotype. In a previous study, QTL mapping assessing the tolerance of maize to low temperatures mainly focused on traits related to the germination process, such as germination rate and germination index (Hu et al., 2016, 2017; Li et al., 2018), while little attention was paid to the growth of shoots and roots after germination.

The response of maize to low temperatures is governed by a complex quantitative genetic traits, controlled by multiple minor genes and easily affected by the environment. Many quantitative trait loci (QTLs) were detected in previous studies, such as a primary QTL on chromosome 6 that is related to cold tolerance. This QTL explains 37.4% of the differences in phenotypes during photo inhibition at low temperatures, and is related to the expression of six other traits (Fracheboud et al., 2004). Five meta-QTLs related to traits associated with the vigor of maize seeds at low temperatures were found on chromosomes 2, 3, 5, and 9 (Shi et al., 2016). Other QTLs, such as *mQTL1-1*, comprised seven QTLs associated with seedling and germination characteristics, including four QTLs under cold temperatures from a population produced by crossing a cold-intolerant (A661) inbred line with a cold-tolerant line (EP42), both of which were related to tolerance to low temperatures at the germination and seedling stages in small populations (Presterl et al., 2007; Rodrı’guez et al., 2014; Li et al., 2018). Previous results demonstrated that QTLs were distributed on 10 chromosomes in maize, and identified no major QTL related to tolerance to low temperatures at the germination stage. This could be because these QTLs are primarily related to the seedling stage. The identification traits (germination rate and germination index) were the same at the germination stage, while some significant traits related to shoots or roots were not included. Previous studies mostly used inbred lines from Europe or America with fewer molecular markers. The strains used in this study, Ye478 and Qi319, are important for breeding in China and respond differently to low temperatures. The 365 recombinant inbred lines (RILs) derived from Ye478 and Qi319 were sequenced using the genotyping by sequencing (GBS) method.

Several genes are associated with low-temperature responses in rice, *Arabidopsis thaliana*, and other plants, including CBFs, MYBs, and MPKs (Li et al., 2017a,b; Wang et al., 2019; Ye et al., 2019). The way in which the genes and related pathways of *A. thaliana* regulate tolerance to low temperatures is relatively clear. For example, PUB25 and PUB26 promote the tolerance of low temperature via degradation of the negative regulator MYB15, which is responsible for cold signaling in *A. thaliana* (Wang et al., 2019). ICE1 phosphorylation mediated by MPK3 and MPK6 regulates ICE1 in a negative manner, and BRASSINOSTEROID-INSENSITIVE2 negatively regulates ICE1 response to cold stress in *A. thaliana* (Li et al., 2017a; Ye et al., 2019). In rice, *COLD1* is a quantitative trait locus that allows *japonica* rice to tolerate frost by activating Ca^2+^ channels in response to low temperatures (Ma et al., 2015). The natural variations of *CTB4a* and *OsMADS57*, the transcription factors of MADS-box, were related to ATP content, while organogenesis genes could enhance the ability of rice to adapt to low temperatures (Zhang et al., 2017b; Chen et al., 2018). Genes related to low-temperature tolerance in maize, such as *ZmCDPK1*, *ZmSEC14p*, and *ZmMPK5* have been detected (Berberich et al., 1999; Kong et al., 2013; Wang et al., 2016). However, the genetic mechanism behind maize tolerance to low temperatures is still unclear. Therefore, it is necessary to perform additional research on how maize tolerates low temperatures.

This study used the genotypic and phenotypic data from 365 maize RILs, which were F_11_ individuals obtained from the self-cross of Ye478*Qi319. The purpose of this study is to (1) analyze QTLs related to low-temperature tolerance using R/qtl software and identify the primary QTL linked by multiple traits, (2) verify the consistent primary QTL linked by multiple traits using the contig substitution mapping method combining genotype and phenotype data of chromosome segment substitution lines (CSSLs), (3) analyze the consistent meta-mQTL data from previous studies, and (4) predict and verify candidate genes in the primary QTL confidence interval.

## Materials and methods

### Plant materials

Total of 365 lines were obtained from a hybrid of two well-known inbred maize strains, the cold-tolerant line Ye478 and the sensitive line Qi319, via single-seed origin of F_11_. The two parent lines had significant differences in eight traits related to cold tolerance, including relative root volume (RRV), relative total length (RTL), relative shoot length (RSL), relative germination rate (RGR), relative root average diameter (RRAD), relative root length (RRL), relative root superficial area (RRSA), and relative simple vigor index (RSVI). Ye478, a dent maize, had an average RGR of 0.845 and an average RSVI of 0.715. In contrast, Qi319, a flint maize, was sensitive to cold, with averages of 0.449 and 0.257 for RGR and RSVI, respectively. Seven CSSLs were selected from the CSSL with donor parent Qi319 and recipient parent Ye478 and were used to verify the QTLs. The details of these seven CSSLs were displayed in Supplementary Table 1 and Supplementary Figure 1.

### Phenotypic evaluation

The seeds from both lines were sterilized with 1% sodium hypochlorite (NaClO) for 5 min and washed with distilled water. They were then soaked in tap water for 6 h and grown in paper rolls at 10°C chambers (treatment) and 25°C chambers (control) for 30 and 6 days in a dark environment, respectively. The chamber was ARC-36L2-E from PERCIVAL. A completely randomized design with three replicates was used for the germination experiment, with each replicate containing 50 seeds. Eight cold-related traits were measured after the seeds were placed in the chambers. The germination rate (GR) was expressed as the percentage of germinating plants out of the total number of seeds used. The root scanner (Epson Perfection V800 scanner) and analysis software (Regent WinRHIZO from Canada) were used to measure the following seven cold-related traits in germinated seedlings: shoot length (SL), root length (RL), root volume (RV), root superficial area (RSA), root average diameter (RAD), simple vigor index (SVI), and total length (TL). The mean of 10 seedlings were used to measure all these seven traits. For evaluation of seed germination ability at low temperatures, the ratio (relative value) of eight traits (RGR, RSL, RRL, RRV, RRSA, RRAD, RSVI, and RTL) were used as indicators for low-temperature tolerance in order to eliminate the differences in genetic background of the different materials. The ratios (relative performance) were calculated as the ratios of the mean values of measurements (*n* = 3) taken under low-temperature treatment conditions and normal temperature conditions (Zhang et al., 2020).

### Phenotypic data analysis

The analysis of variance (ANOVA), as well as QTL mapping, was performed using the mean of all replicates. A combined ANOVA spanning several environments with the Mixed Linear Model procedure (PROC MLM) and Statistical Analysis System (SAS) software version 9.2 (SAS Institute, Cary NC, United States, 2009) were performed, which allowed us to approximate the variance. Linear regressions with significance levels of *P* = 0.05 were used to calculate Pearson’s correlation coefficients (*r*) for each characteristic. Pearson correlation coefficients (*r*) between different traits were determined by linear regressions at the significance level *p* = 0.05, and calculated using SPSS20.0 (IBM corp., Armonk, NY, United States). The following equation was used to calculate the coefficients of variation (CV, %): CV = *s*/*x*. In this equation, “*x*” equals each trait’s mean in a population and “*s*” is equals the standard deviation.

### Mapping linkages

The GBS technology (The original genotypic datasets have become public in the NCBI database^2^ under the accession PRJNA627044), were used with an Illumina 2500 platform and methods previously described to characterize the RIL population (Zhou et al., 2016). Total of 86,257 SNPs were identified and generated an ultra-high density linkage map using 4,602 bin markers (100-Kb intervals with no recombination events). The map had a total genetic distance of 1,533.72 cM, with an average distance of 0.33 cM between markers (Zhou et al., 2016). Composition-interval mapping (CIM) was used to identify the QTLs in the *R/qtl* package. The threshold of the logarithm of the odds (LOD) scores were determined using 1,000 permutations and a significance level of *p* = 0.05. These were used to evaluate the effects of the QTL. The QTLs with LOD figures higher than the threshold, which was 2.5, warranted additional study. The *ftqtl* function from the *R/qtl* package was used to assess the phenotypic variation of the identified QTLs. The consistent QTLs influencing multiple traits were named with the initial “c,” which represents consistent, and the numbers in the name indicate chromosome and number.

### Chromosome segment substitution lines materials and genotypic data screening

The population with 180 CSSLs were constructed with Ye478 as the female parent and Qi319 as the male parent. These CSSLs were selected by backcrossing and marker-assisted selection technology by SSR and InDel marker encryption (Wang et al., 2018b). In this study, seven CSSLs with segments substitution of Qi319 in the major QTL of low-temperature tolerance were selected and used. In the seven CSSLs, six lines (CL6, CL9, CL14, CL17, CL18, and CL173) contain only one homozygous genomic segment of Qi319, respectively. However, line CL174 contain two genomic segments including a homozygous segment of Qi319 and a hybrid segment. The background recovery rates in seven CSSLs were all more than 96% (Supplementary Figure 1). The GR of each of the seven CSSLs were detected and the RGR was calculated to verify the accuracy of identification of the main effect QTL in the RIL population and to reduce the confidence interval of the QTL.

### Meta-QTL analysis

The mapping information of 76 QTLs related to cold tolerance in the germination stage of maize were collected from recently published papers and our own research. This information included markers, traits, names, chromosomes, and Linkage Group selection (LGs). The original QTL maps to the reference map IBM2 2008 Neighbors were compared, which shares enough markers with other maps to make an accurate projection. As such, the IBM2 2008 Neighbors integrated QTLs from other populations. A homothetic function were used to project the QTLs to the reference map by estimating the most likely position and CI. The projected QTLs related to cold tolerance were used to construct a consensus map of cold-related traits with the BioMercator ver.2.1 software (Arcade et al., 2004). A meta-analysis using this software from different independent experiments, QTLs associated with similar LGs, and QTLs at neighboring intervals to generate an optimal QTL. While QTLs provided five different models, the best QTL model was the Akaike Information Criteria (AIC). This was considered the optimal QTL. The optimal QTL was close to the smallest AIC, while the mean R2 values of the original QTLs in the region explained the variance of the optimal QTL. The Meta-QTLs were named with the initial “m,” which represent meta. The consistent QTLs and Meta-QTLs were named with the initial “Lt,” which represent low-temperature, and the numbers in the name indicate chromosome and number.

### Candidate gene prediction and identification

Based on the comprehensive analysis results of RIL population, CSSLs and Meta-QTL analysis, combined with MaizeGDB^3^, NCBI database (see Text Footnote 2), and UniPort^4^, the gene annotation function of B73 (B73 RefGen_v3) was searched for this major QTL segment. Well-annotated genes related to low-temperature tolerance and other abiotic stresses were obtained from *A. thaliana*, *Sorghum bicolor*, and *Oryza sativa*. Two genes were selected from our confident QTL interval in order to validate them with quantitative PCR (qPCR). A total of six maize inbred lines of Ye478 (tolerant), Qi319 (sensitive), ZYQ219 (tolerant), ZYQ011 (sensitive), CL082 (tolerant), and CL018 (sensitive) were used as test materials. Their relative germination rate phenotypes were listed in Supplementary Table 2. Two groups of seeds were soaked in tap water for 6 h and then grown at 10°C/25°C for 2 and 4 h in chambers, respectively. From each replication, 10 seeds were ground in liquid nitrogen to extract the total RNA with *TransZol*™ Up Plus RNA Kit [TransGen Biotech (Beijing, China)]. They were then subjected to reverse transcription reaction of cDNA via RT MasterMix [TransGen Biotech (Beijing, China)], while qPCR analysis was performed on a *TransStart*^®^ Tip Green qPCR SuperMix kit [TransGen Biotech (Beijing, China)]. Supplementary Table 3 displays the primers used for qPCR. The Actin gene from maize was used for an internal control, and the mean of three replications was used to express the final gene. The candidate gene relative expression level was calculated using the 2^–ΔΔ*Ct*^ analytical method. And the gene expression was translated to log_2_(fold change).

## Results

### Phenotypic traits relating to tolerance of low temperatures

The descriptive statistics of the morphological traits at the germination stage in the RIL populations were displayed in Table 1. Two parental inbred lines showed highly significant differences (*P* < 0.01) in seven traits (RGR, RSL, RRL, RTL, RRSA, RRAD, RRV, and RSVI), except RRAD that showed a significant difference (*P* < 0.05). All traits were normally and continuously distributed in all 365 RILs, which also displayed quantitative inheritance. For example, RGR ranged from 0.088 to 0.993 and had a mean of 0.680 in the RIL population, and RSVI ranged from 0.036 to 0.765 and had a mean of 0.343. RSVI had the highest CV (0.365) in the RIL population, while RRAD (0.035) had the lowest CV. Within the RIL population, the broad-sense heritability (*H*^2^) related to eight characteristics related to the germination stages spanned from 0.824 for RSVI to 0.907 for RRL (Supplementary Table 4).

**TABLE 1:** QTLs identified for eight maize cold-related traits.

No.	QTL name	Chr.	Flanking markers	Interval (Mb)	Physical length (Mb)	LOD	PVE	ADD	Phenotype
(1)	*qRSVI1-1*	1	mk234-mk243	63.85–68.45	4.60	5.64	7.80	−0.25	RSVI
(2)	*qRGR1-1*	1	mk235-mk243	64.20–68.45	4.25	11.02	13.52	−0.14	RGR
(3)	*qRGR1-2*	1	mk445-mk466	197.10–201.95	4.85	5.03	8.05	−0.11	RGR
(4)	*qRRSA1-1*	1	mk445-mk465	197.10–201.60	4.50	5.17	5.18	−0.23	RRSA
(5)	*qRSL1-1*	1	mk446-mk466	197.75–201.95	4.20	4.19	6.06	−0.08	RSL
(6)	*qRRL1-1*	1	mk446-mk466	197.75–201.95	4.20	19.71	17.01	−0.42	RRL
(7)	*qRTL1-1*	1	mk446-mk466	197.75–201.95	4.20	19.71	20.36	−0.23	RTL
(8)	*qRRV1-1*	1	mk446-mk467	197.75–202.30	4.55	6.97	8.44	−0.33	RRV
(9)	*qRSVI1-2*	1	mk446-mk467	197.75–202.30	4.55	20.09	25.96	−0.16	RSVI
(10)	*qRRL3-1*	3	mk1225-mk1232	7.75–8.65	0.90	2.88	3.58	−0.17	RRL
(11)	*qRTL3-1*	3	mk1226-mk1227	7.90–8.05	0.15	2.69	3.79	−0.07	RTL
(12)	*qRSL3-1*	3	mk1295-mk1313	21.05–25.65	4.60	4.81	4.23	−0.08	RSL
(13)	*qRSL3-2*	3	mk1472-mk1489	164.10–169.00	4.90	5.65	6.47	−0.06	RSL
(14)	*qRRAD3-1*	3	mk1611-mk1639	206.80–211.75	4.95	7.40	8.37	0.01	RRAD
(15)	*qRRL7-1*	7	mk3161-mk3180	1.45–4.85	3.40	7.22	7.14	−0.25	RRL
(16)	*qRTL7-1*	7	mk3177-mk3193	3.65–8.40	4.75	6.46	6.39	−0.13	RTL
(17)	*qRSVI7-1*	7	mk3178-mk3194	3.80–8.75	4.95	3.86	3.59	−0.09	RSVI
(18)	*qRRAD8-1*	8	mk3839-mk3864	158.10–162.15	4.05	6.00	5.54	0.02	RRAD
(19)	*qRGR9-1*	9	mk3961-mk3984	1.35–5.75	4.40	4.90	5.07	−0.08	RGR
(20)	*qRGR10-1*	10	mk4539-mk4546	139.10–140.75	1.65	3.61	4.56	0.08	RGR

**FIGURE 1:**
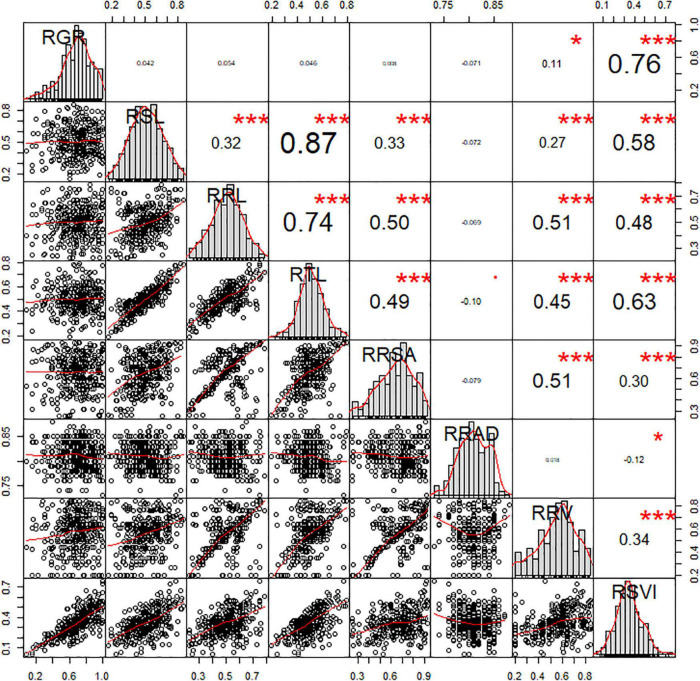
Correlation analysis of the RIL population related to low-temperature tolerance traits. The frequency distribution of each trait is shown on a central diagonal in the form of a histogram. Scatter plots of between each pair of traits are shown in the areas below the diagonal, and numerical correlation coefficients between each pair of traits are shown in the areas above in the diagonal. * and ^***^ indicate significance at *p* < 0.05 and *p* < 0.001, respectively.

Of the eight morphological traits relating to the germination stage analyzed in this study, several significant correlations were observed. The significant correlations between RSVI and six other traits were also been observed, which played an important part in low-temperature resistance at the germination stage. The r values of these correlations were 0.76, 0.58, 0.48, 0.63, 0.30, and 0.34 for RGR, RSL, RRL, RTL, RRSA, and RRV, respectively. RRV displayed significant positive correlations with RRL (*r* = 0.51, *P* < 0.01), RSL (*r* = 0.27, *P* < 0.01), RTL (*r* = 0.45, *P* < 0.01), and RRSA (*r* = 0.51, *P* < 0.01). RRSA displayed significant positive correlations with RSL (*r* = 0.33, *P* < 0.01), RRL (*r* = 0.50, *P* < 0.01), and RTL (*r* = 0.49, *P* < 0.01). Three traits (RRL, RSL, and RTL) displayed significant positive correlations with each other (*P* < 0.01) (Figure 1).

### Quantitative trait locis identification of low-temperature tolerance

Total of 19 QTLs were associated with eight traits in the control group, while 2, 4, 4, 2, 1, 2, 2, and 2 QTLs were associated with normal germination rate (NGR), normal shoot length (NSL), normal root length (NRL), normal total length (NTL), normal root superficial area (NRSA), normal root average diameter (NRAD), normal root volume (NRV), and normal simple vigor index (NSVI), respectively. These QTLs were detected on chromosomes 1, 2, 3, 4, 5, 7, 8, and 10, with LOD values ranging from 2.65 to 6.34, and the physical lengths from 0.25 to 2.80 Mb (Supplementary Figure 2 and Supplementary Table 5).

**FIGURE 2:**
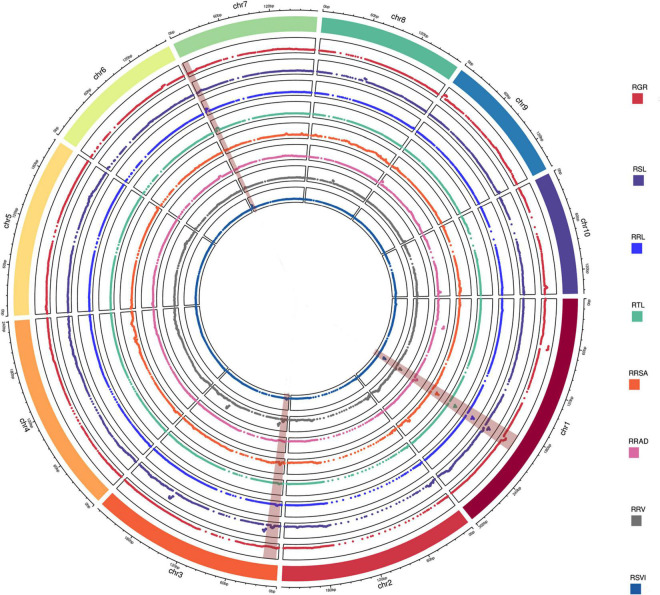
QTL analysis in the RIL population related to low-temperature tolerance traits. The outermost box with scale represents the 10 maize chromosomes. For each trait, different colors represent the eight different traits related to low-temperature tolerance (RGR, RSL, RRL, RTL, RRSA, RRAD, RRV, and RSVI). The red backgrounds of chromosomes 1, 3, and 7 represent consistent QTLs.

A total of 20 QTLs were associated with eight relative traits, while 4, 3, 3, 3, 1, 2, 1, and 3 QTLs were associated with RGR, RSL, RRL, RTL, RRSA, RRAD, RRV, and RSVI (Figure 2), respectively. These QTLs were detected on chromosomes 1, 3, 7, 8, 9, and 10, with LOD values ranging from 2.69 to 20.09. Of these QTLs, more than 85% had a negative additive effect. This suggests that the parent Ye478’s alleles resulted in higher phenotypic values. When assessed against the B73 RefGen_v3 genome, the confidence intervals for these QTLs averaged 3.93 Mb and ranged from 0.15 to 4.95 Mb. The individual QTLs explained 8.56% of the phenotypic variations, ranging from 3.58% (RRL, *qRRL3-1*) to 25.96% (RSVI, *qRSVI1-2*) for eight traits (Table 1).

**FIGURE 3:**
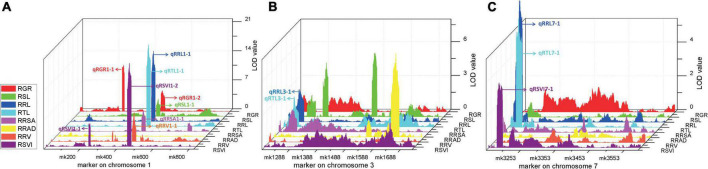
QTL map of consistent segments related to multiple low-temperature tolerance traits in the RIL population during germination. **(A)** QTL of eight phenotypes on chromosome 1, **(B)** QTL of eight phenotypes on chromosome 3, **(C)** QTL of eight phenotypes on chromosome 7. For each trait, different colors represent the eight different traits related to low-temperature tolerance (RGR, RSL, RRL, RTL, RRSA, RRAD, RRV, and RSVI).

Four stable or consistent QTLs were detected for at least two traits. Two consistent QTLs influencing multiple traits were found on chromosome 1. The *cQTL1-2* region (position 197.10–202.30 Mb on chromosome 1) possessed seven QTLs related to germination, with consistent QTLs of RGR, RSL, RRL, RTL, RRSA, RRV, and RSVI. These explained phenotypic variances from 5.18 to 25.96%, suggesting a close genetic relationship between the roots of the germinates and the indicators, possibly due to pleiotropy. *cQTL1-1* was found on chromosome 1 at position 63.85 to 68.45 Mb. It accounted for 7.80 and 13.52% of the respective total phenotypic variance for RSV and RGR (Figure 3A). The *cQTL3-1* with *qRRL3-1* and *qRTL3-1* were detected on chromosome 3 (Figure 3B). One consistent QTL (*cQRL7-1*) on chromosome 7, from 1.45 to 8.75, included three QTLs of *qRRL7-1*, *qRTL7-1*, and *qRSVI7-1*. These phenotypic variances ranged from 3.59 to 7.41% (Figure 3C).

### Verification and fine mapping of quantitative trait locis with chromosome segment substitution lines

**FIGURE 4:**
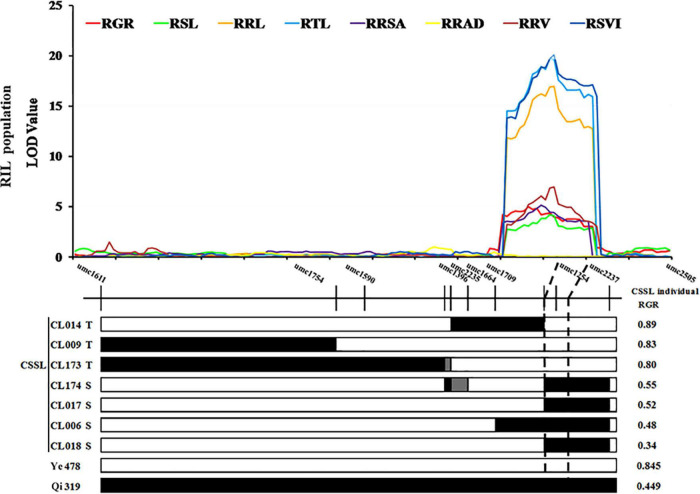
*cQTL1-2* fine mapping in CSSL lines. White represents the homozygous segment of Ye478, black is the homozygous segment of Qi319, gray is the hybrid segment. T- material of tolerant line, S- material of sensitive line. For each trait, different colors of LOD value represent the eight different traits related to low-temperature tolerance (RGR, RSL, RRL, RTL, RRSA, RRAD, RRV, and RSVI) of the RIL population.

Two SSR markers umc1254 and umc2237 were added in *cQTL1-2* (chr1: 197.10–202.30 Mb) of a CSSLs population with 180 families which was constructed with Ye478 as the female parent and Qi319 as the male parent. In these CSSLs, the genotypes of CL174, CL017, CL006, and CL018 introduced Qi319 fragment from markers umc1254 to umc2237. However, the genotypes of CL014, CL009, and CL173 were still from the recurrent parent Ye478 fragment, and the imported Qi319 fragment was located near *cQTL1-2* (Supplementary Table 1 and Supplementary Figure 1). To verify the consistency and narrow the consistent QTL range, seven CSSLs were analyzed with two major QTLs, which were constructed with the same parental inbred line. The RGR trait was used to verify *cQTL1-2*. The RGRs of the segment substitution lines CL174, CL017, CL006, and CL018, which were substituted with Qi319, were changed from 0.89 to 0.34–0.55. These were significantly different from the RGRs of Ye478 (*P* < 0.001). *cQTL1-2*, which controlled ability to germinate at low temperatures, was between the umc1254 and umc2237 markers (200,400,148–206,699,769 bp). Combined with *cQTL1-2*, the major QTL was from markers umc1254 and umc2505 (200,400,148–202,300,000 bp), with a confidence interval of 1,899,852 bp (Figure 4).

### Meta-analysis verification of consistent QTL

**FIGURE 5:**
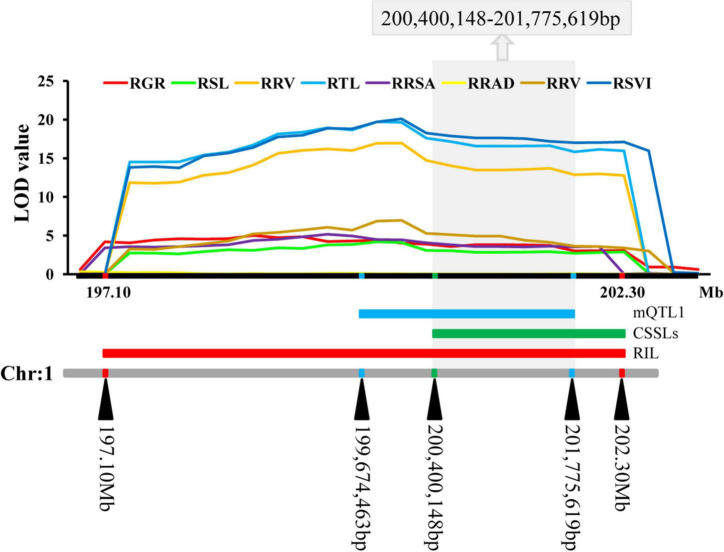
The consistent segments during the germination related to low-temperature. For each trait, different colors of LOD value represent the eight different traits related to low-temperature tolerance (RGR, RSL, RRL, RTL, RRSA, RRAD, RRV, and RSVI).

The QTLs were distributed on all the ten chromosomes of maize in clusters of distribution (Supplementary Figure 3 and Supplementary Table 6). The most QTLs (22) were detected on chromosome 1, while six QTLs were detected on chromosome 2, nine were detected on chromosome 3, four were detected on chromosome 4, seven were detected on chromosome 5, the least (three) were detected on chromosome 6, four were detected on chromosome 7, four were detected on chromosome 8, 12 were detected on chromosome 9, and five were detected on chromosome 10. These QTLs explained between 0.62 and 39.44% of phenotypic variation. A meta-QTL (*mQTL1*) was detected on chromosome 1, with 11 QTLs. These QTLs were co-located and distributed in clusters. The *mQTL1* was located on chromosome 1, from 199,674,463 to 201,775,619 bp in bin 1.06–1.07 with molecular markers of ereb172 and tena2. Combined with the results of *cQTL1-2* and *mQTL1*, the consistent major low-temperature tolerance QTL (*LtQTL1-1*), which controls ability to germinate in maize, was on bin 1.06–1.07 on chromosome 1 at a range of 200,400,148–201,775,619 bp (Figure 5).

### Quantitative PCR validation for candidate genes

**FIGURE 6:**
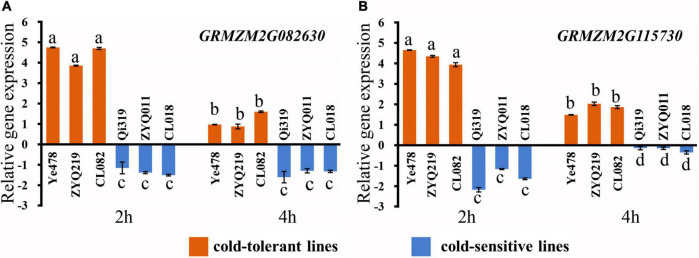
Relative expression of two candidate genes. **(A)** Relative expression of the candidate gene *GRMZM2G082630*, **(B)** relative expression of the candidate gene *GRMZM2G115730*. Different small letters within a gene indicate significant differences between the materials.

Referring to B73 in MaizeGDB (see Text Footnote 3) RefGen_v3 genome annotation information, there were 66 genes in the *LtQTL1-1* confidence interval. Of these, 26 were annotated to be mainly related to transport, stress response, signal transduction, catalytic activity, binding activity, and cell components. Two candidate genes (*GRMZM2G082630* and *GRMZM2G115730*) within *LtQTL1-1* were similar to the genes relating to low-temperature adaptation published by BLAST analysis (Supplementary Table 7). qRT-PCR was used to confirm the levels of expression and confirm these two candidate genes. Of the six maize materials, there were two parental inbred lines, a low-temperature resistant and sensitive line, from RILs and CSSLs. These were used to detect the level of genetic expression under low temperatures. These two genes displayed significant positive expression levels in the low-temperature resistant lines (Ye478, ZYQ219m, and CL082) and negative expression levels in the low-temperature sensitive lines (Qi319, ZYQ011m, and CL018), 2 and 4 h following exposure to cold temperatures. *GRMZM2G082630* and *GRMZM2G115730* expression levels differed between the resistant and sensitive lines; further, expression levels of the two genes also showed significant differences between 2 and 4 h (*p* < 0.01) (Figure 6).

## Discussion

### Identification of traits related to low-temperature tolerance

Recent studies on plant stress resistance suggested that when plants are subjected to abiotic stress, their root structure changes to improve stress tolerance (Bao et al., 2014; Robbins and Dinneny, 2015). Therefore, root characteristics are an important measure of stress resistance. Research assessing the QTL mapping of the ability of maize to tolerate low temperature has mainly focused on traits related to germination, such as the germination rate and germination index (Hu et al., 2017; Li et al., 2018). Root traits such as length, fresh weight, dry weight, and water content were primarily measured at the seedling stage (Jompuk et al., 2005; Presterl et al., 2007; Rodrı’guez et al., 2014). Little attention was paid to root and shoot growth after the germination period; however, the root and shoot characteristics during the germination period help regulate the growth of maize (Hund et al., 2004). Thus, in the current study, eight traits including RGR, RSL, RRL, RTL, RRSA, RRAD, RRV, and RSVI were used to detect the QTLs of low temperatures during the germination period in maize. The results showed that RGR was significantly positively correlated with RSVI, while RSVI was also significantly positively correlated with RSL, RRL, RTL, RRSA, and RRV. However, the correlation between RRAD and other traits were not significant. The results of QTL mapping demonstrated that RGR, RSL, RRL, RTL, RRSA, RRV, and RSVI were all mapped to the main QTL segment (197.10–202.30 Mb on chromosome 1), and the contribution rate of each phenotype in this major QTL segment ranged from 5.18 to 25.96%. This indicates that the shoot and root traits were closely related to the species during seed germination. The seed germination rate should also be considered in QTL mapping, and the roots and shoots at the germination stage are also related to the stress response of low-temperature tolerance in maize. Of the 20 QTLs mapped, root-related traits were important. When 15 QTLs were mapped, the phenotypic contribution rate ranged from 3.58 to 25.96%. Therefore, the root system plays a vital role in the adaptation of plants to stress conditions. Low temperatures can weaken, inhibit, and reduce root length, volume, and dry weight (Hodges et al., 1995; Bhosale et al., 2007; Rácz et al., 2007; Frascaroli and Landi, 2013, 2016). The results of this experiment were consistent with those of previous studies.

### The advantages of bin map or high-throughput sequencing in QTL analysis

In plants, the bin map genetic linkage map obtained by high-throughput sequencing technology has a high-density and small QTL interval, and is widely used. Therefore, used a 2,500-locus bin map of the homologous group 5 in wheat to better understand the distribution and collinearity of its genes with that of rice (Linkiewicz et al., 2004). The researchers generated a high-resolution genetic map of the PmAS846 locus in order to assess the resistance of wheat to powdery mildew (Xue et al., 2012). The QTLs related to anaerobic germination tolerance and salt stress at early seedling stages in rice were also identified via high-density bin genetic map (Yang et al., 2019; Amoah et al., 2020). In maize, some RILs were constructed to identify QTLs and genes. One example is a set of 204 RILs (with parents Zheng58 and Chang7-2), which was the widely adopted Chinese hybrid ZD958 (Song et al., 2016). From this, 199 F_2_ offspring were obtained by crossing the varieties SG-7 and SG-5 and genotyping them via GBS (Su et al., 2017), as well as a set of RILs derived from inbred lines Ye478 and Qi319 (Zhou et al., 2016). QTLs relating to yield, plant architecture, and seedling root system architecture traits were all mapped using the high-density linkage map (Courtial et al., 2013; Chen et al., 2016; Song et al., 2016; Su et al., 2017; Zhang et al., 2017a; Wang et al., 2018a). In this study, an ultra-high-density genetic linkage map (with 4,602 bin markers) and GBS high-throughput sequencing were used to perform QTL mapping. The low-temperature tolerance of these QTLs were mapped at the germination period to a range of 0.90-4.95Mb. The range of the mapped QTLs was smaller than others. The results were also stable, with several traits were located together, and the narrowed interval was suitable for further prediction of candidate genes.

### The importance of chromosome segment substitution lines in QTL mapping

Chromosome segment substitution lines are one of the best methods of QTL mapping and are widely used in QTL analysis of agronomic and stress resistance traits in plants. CSSLs are most widely used in rice and for QTL mapping in maize. QTLs relating to the resistance of multiple diseases in maize were identified by CSSLs (Lopez-Zuniga et al., 2019). A set of 184 CSSLs and their inbred lines (Zheng58 and Xun9058) were used to identify maize kernel traits (Wang et al., 2018c). A set of 130 CSSLs were constructed with (donor parent Nongxi531 and recipient parent H21) to perform a QTL analysis of the number of kernel rows in maize. This demonstrated their true expression in different environments (Li et al., 2014). The CSSLs derived from Qi319 as donor and Ye478 were used to validate *qNCLB7.02*, which was the novel QTL related to resistance to the northern corn leaf (NCLB) (Wang et al., 2018b). However, there have yet been few reports on applying CSSL materials when mapping the QTLs related to maize tolerance of low temperatures. In this study, the RIL population was first constructed by Ye478*Qi319 to map QTLs related to tolerance to low temperatures in maize, in order to determine one or more traits. The linked consistent QTL segment is located on 197.10–202.30 Mb of chromosome 1, and the physical distance is 5.20 Mb. This QTL segment is named *cQTL1-2*. The CSSLs with the donor parent-introduced fragments near the *cQTL1-2* segment were subjected to phenotypic testing and the contig substitution mapping method was used to verify the accuracy of the *cQTL1-2* positioning of the RIL population. This was reduced to 200,400,148–202,300,000 bp, with a physical distance of about 1.9 Mb.

### Comparative analysis of quantitative trait locis relating to maize tolerance of low temperatures

In this study, a linkage analysis of the RIL population were performed on eight low-temperature tolerance related traits and mapped a total of 20 QTLs located on different chromosomes. Of them, four were new QTLs that had not been previously mapped: *qRSL3-2*, *qRRAD8-1*, *qRGR9-1*, and *qRGR10-1*. The remaining 16 QTLs overlapped with QTLs known to be related to low-temperature tolerance, and this study narrowed the confidence interval of their positioning. This study assessed the QTLs of RGR, RSL, RRL, RTL, RRSA, RRV, and RSVI: *qRGR1-2*, *qRSL1-1*, *qRRL1-1*, *qRTL1-1*, *qRRSA1-1*, *qRRV1-1*, and *qRSVI1-2*. The mapped *cQTL1-2* segment of chromosome 1 (197.10–202.30 Mb) was consistent with QTLs (58.66 Mb) for shoot length, root length, and total length (Li et al., 2018), the phosphoric acid QTLs for enolpyruvate carboxylase activity (70 Mb) (Leipner and Mayer, 2008), the QTLs for φPSII traits (43 Mb) (Fracheboud et al., 2004), and the SNP related to chlorophyll content (PZE-101159230) (Revilla et al., 2016). In previous studies, the QTL was narrowed to 5.2 Mb (chr1: 197.10–202.30 Mb) from 43 to 70 Mb. In this experiment, the CSSLs were used to reduce the *cQTL1-2* to approximately 1.9 Mb, using the contig substitution mapping method. This was used along with a meta-analysis to verify the accuracy of *cQTL1-2* and further reduce it to 1.38 Mb, which was named *LtQTL1-1* (200,400,148–201,775,619 bp). *LtQTL1-1* is the major QTL linked to multiple low-temperature tolerance traits and had a phenotypic contribution rate from 5.18 to 25.96%. Additionally, the SNP related to the chlorophyll content at low temperatures (PZE-101159230) (Revilla et al., 2016) was also located in our major QTL. The SNP-31 associated with relative water content at low temperatures was located in the *qRSL3-1* of our QTLs (Huang et al., 2013). The SNP (S7_1956860) associated with the relative number of days when germination rate reaches 50% was located in the QTL *qRRL7-1* (Hu et al., 2017).

### Molecular function of two candidate genes

*GRMZM2G082630* was the protein that codes for superfamily of basic Helix Loop Helix (bHLH) domain. The bHLH proteins were transcriptional regulators, and members of this superfamily with two functionally distinct regions, which were highly conserved: a basic DNA binding region and a helix-loop-helix (HLH) region. The characteristics of superfamily of bHLHs in *A. thaliana* were play important function of stress responses, light signal transduction, plant growth and development (Friedrichsen et al., 2002; Abe et al., 2003; Castelain et al., 2012; Liu et al., 2013; Yao et al., 2018). They were also participate in the crosstalk of hormone signaling, such as jasmonic acid (JA), salicylic acid (SA), abscisic acid (ABA), brassinosteroid (BR), and ethylene (ET) (Murre et al., 1989; Heim et al., 2003; Pires and Dolan, 2010; Feller et al., 2011), and they are critical for survival in the environment (Hao et al., 2021). Previous studies show that the homologous bHLH genes *bhlh068* of *O. sativa* and *bHLH112* of *A. thaliana* are important regulatory factor to response to salt stress (Chen et al., 2017). The *Nicotiana tabacum* plants which overexpressing *Ntbhlh123* can enhanced resistance of under low-temperature (Zhao et al., 2018). The genes *SbbHLH134*, *SbbHLH110*, and *SbbHLH101*, which have bHLH domain in *S. bicolor*, also can regulate flower and fruit development (Fan et al., 2021). *GRMZM2G115730* was encoded by the evolutionarily conserved protein with the Epsin N-Terminal Homology (ENTH) domain. The domain was a portion of structurally related ENTH, ANTH, or VHS domain in the N-terminal region and a variable C-terminal region, with the functions of transport vesicle (Feng et al., 2022). The ENTH domain protein family taken part in numerous plant processes, such as, response to abiotic stress, growth of pollen tube, growth and development. This domain could be detected in more than 30 *A. thaliana* proteins, which was involved in clathrin-related endomembrane trafficking of plants (Zouhar and Sauer, 2014). *OsMIP1* encoded a putative transmembrane protein with an ENTH/ANTH/VHS domain, and could respond to NaCl, PEG, and other abiotic stresses (Wang et al., 2017). ENTH family proteins might also plays an important role in the regulation of abiotic stress such as low-temperature. Therefore, two candidate genes, *GRMZM2G082630* and *GRMZM2G115730*, were screened for qRT-PCR validation.

## Conclusion

This study performed QTL mapping of the 365 RILs which obtained from crossing Qi319 and Ye478. Major QTL was verified by seven CSSLs derived from Ye478*Qi319. And a meta-QTL analysis were performed of the ability of maize to tolerate low temperatures at the germination stage. The QTL *LtQTL1-1* related to tolerance of low temperatures at the germination stage was detected on bin1.06–1.07 of chromosome 1, at a confidence interval of between 200,400,148 and 201,775,619 bp. In this interval, the relative expression of the candidate genes *GRMZM2G082630* and *GRMZM2G115730* were significantly different (*p* < 0.01) from that of materials with different low-temperature tolerances. Both genes were up-regulated in low-temperature-tolerant varieties and down-regulated in low-temperature-sensitive varieties.

## Data availability statement

The datasets presented in this study can be found in online repositories. The names of the repository/repositories and accession number(s) can be found below: https://www.ncbi.nlm.nih.gov/, PRJNA627044.

## Author contributions

YZ, QL, and DW performed the experiments and wrote the manuscript. JM, XL, HD, LZ, LD, XJL, XZ, and ZZ took participate in the experiments. ZW, JW, and YZ designed the experiments and revised the manuscript. All authors read and approved the manuscript.

## Footnotes

1
http://faostat.fao.org/

2
https://www.ncbi.nlm.nih.gov/

3
https://www.maizegdb.org/

4
https://www.uniprot.org/
