# Supplementary material for: Using a high density bin map to analyze quantitative trait locis of germination ability of maize at low temperatures
Source: Front Plant Sci. 2022 Aug 22;13:978941. doi: 10.3389/fpls.2022.978941 (PMC9441762; doi:10.3389/fpls.2022.978941)
Supplement: Supplementary file 1 [file Data_Sheet_1.zip › Supplement Figure-0726.pdf]

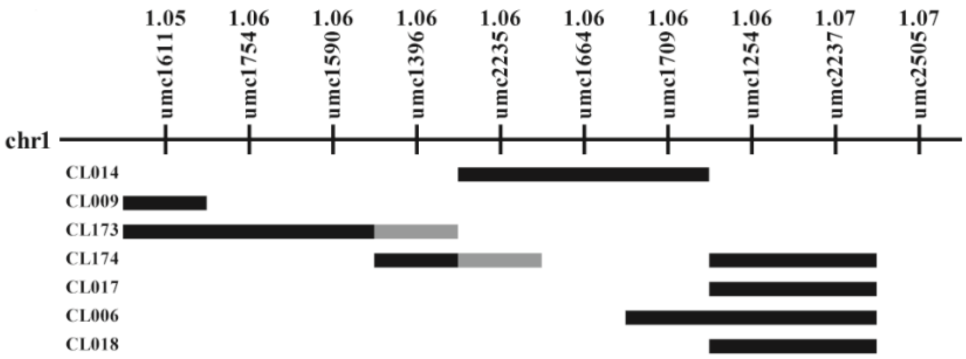

Supplementary Figure 1 Genotype of seven lines of CSSLs

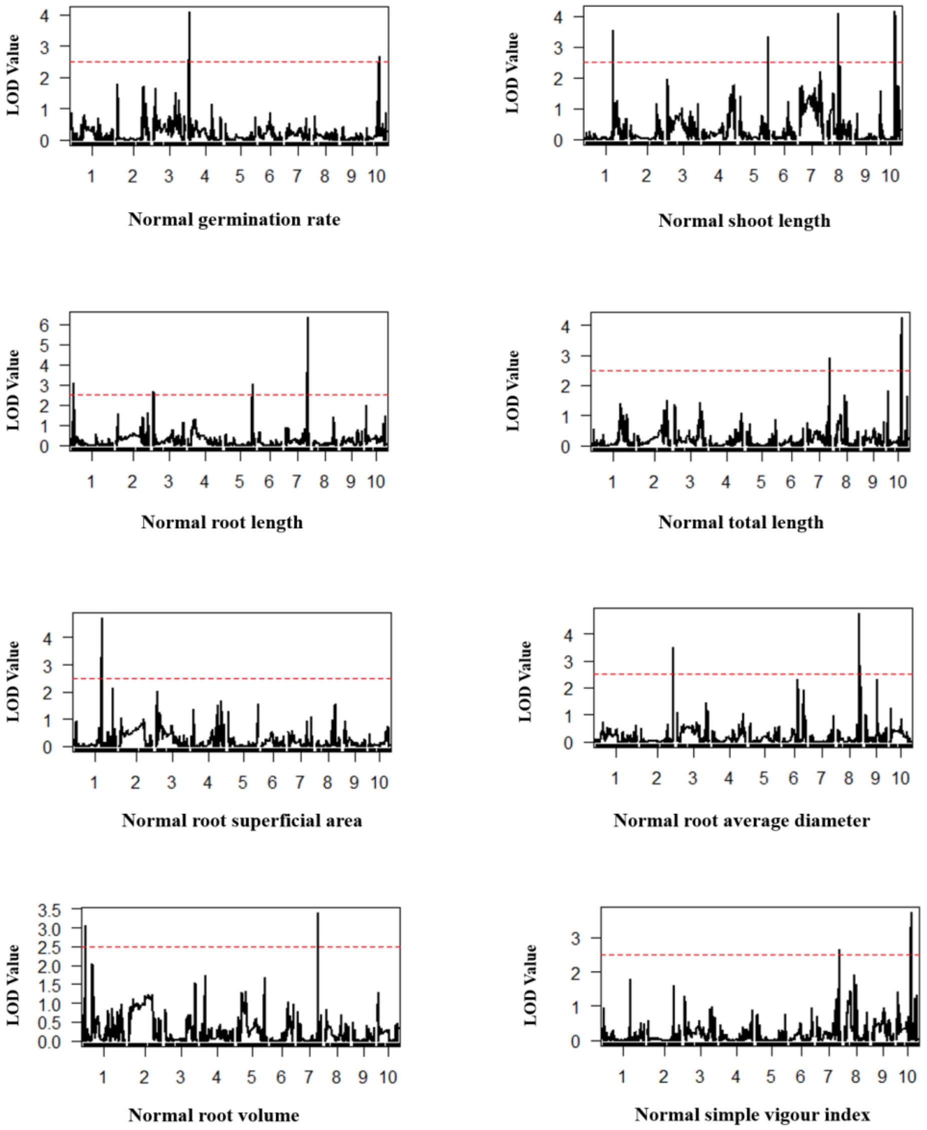

Supplementary Figure 2 QTL analysis in the RIL population the normal traits.

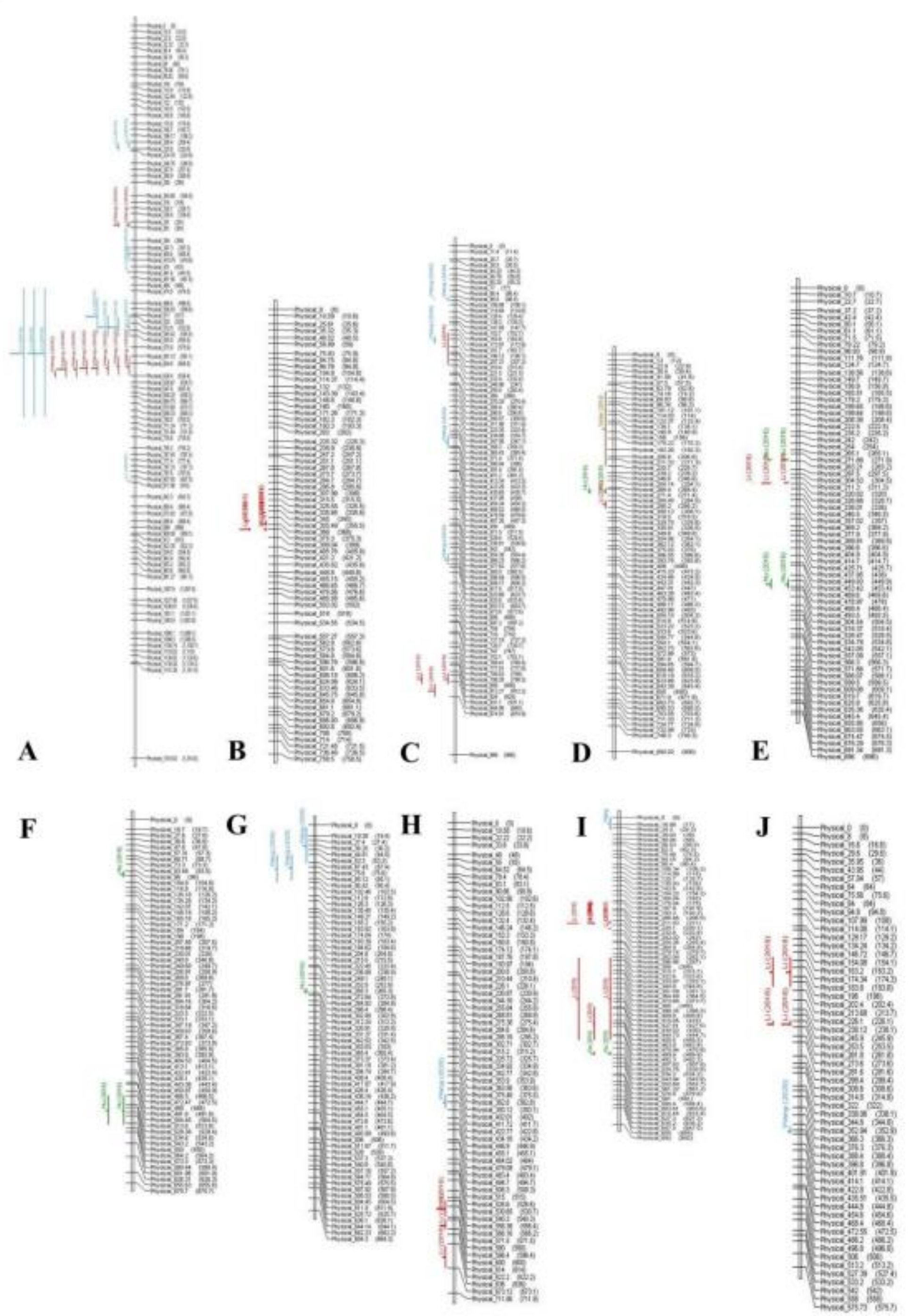

Supplementary Figure 3 Integrated map of QTLs for low-temperature tolerance related traits in maize.
